# HEALTHCARE WORKERS’ PERSPECTIVES ON AI-ENABLED ROBOTS USE IN LONG-TERM CARE: A SCOPING REVIEW

**DOI:** 10.1093/geroni/igad104.3385

**Published:** 2023-12-21

**Authors:** Lillian Hung, Karen Lok Yi Wong, Joey Wong, JuYoung Park, Hadil Alfares, Yong Zhao, Hossein Mousavi, Hui Zhao

**Affiliations:** University of British Columbia, Vancouver, British Columbia, Canada; University of British Columbia, Vancouver, British Columbia, Canada; University of British Columbia, Vancouver, British Columbia, Canada; Florida Atlantic University, Boca Raton, Florida, United States; University of British Columbia, Vancouver, British Columbia, Canada; University of British Columbia, Vancouver, British Columbia, Canada; University of British Columbia, Vancouver, British Columbia, Canada; James Madison University, Harrisonburg, Virginia, United States

## Abstract

Artificial intelligence (AI) enabled robots are increasingly implemented in long-term care homes (LTC). However, the views of LTC staff on these robots remain largely unexplored. Our scoping review delves into the staff’s perceptions, outlining the advantages and challenges of using AI robots in LTC settings. Using the Joanna Briggs Institute’s methodology, we screened 86 articles from 2013 to 2023, with 35 fitting our criteria. Our analysis was informed by McCormack’s Person-centred Care Practice (PCP) Framework and the Consolidated Framework for Implementation Research (CFIR). We identified five key barriers: 1) the complexity of the robot, 2) potential job losses and increased workload, 3) concerns about safety and efficacy, 4) risk of depersonalized care, and 5) a lack of supportive regulations and resources in LTC facilities. To address these challenges, we recommend strategies: a) staff training, b) clarifying robot benefits to staff, c) demonstrating how robots can fulfill resident needs, d) implementing ethical guidelines, and e) aligning robot use with LTC policies while ensuring resource availability. In conclusion, partnership is required among healthcare workers, organizational leaders, robot developers, and researchers; they should not work in silos. More research is needed to explore how to facilitate effective partnerships.

